# A clinical and cost-effectiveness trial of a parent group intervention to manage challenging restricted and repetitive behaviours in young children with autism spectrum disorder: study protocol for a randomised controlled trial

**DOI:** 10.1186/s13063-021-05175-y

**Published:** 2021-04-01

**Authors:** Victoria Grahame, Linda Dixon, Sue Fletcher-Watson, Deborah Garland, Magdalena Glod, Jane Goodwin, Zoe Grayson, Saoirse Heron, Emma Honey, Rebecca Iversen, Adetayo S. Kasim, Ashleigh Kernohan, Ehsan Kharatikoopaei, Ann Le Couteur, Leila Mackie, Ayesha Mathias, Helen Probert, Deborah Riby, Priyanka Rob, Leanne Rogan, Sarah Thompson, Luke Vale, Eamonn Walls, Elspeth Imogen Webb, Christopher Weetman, Faye Wolstenhulme, Ruth Wood, Jacqui Rodgers

**Affiliations:** 1 Cumbria, Northumberland, Tyne and Wear NHS Foundation Trust, Complex Neurodevelopmental Disorder Service (CNDS), Walkergate Park, Benfield Road, Newcastle upon Tyne, NE6 4QD UK; 2 Salvesen Mindroom Research Centre, The University of Edinburgh, Kennedy Tower, Morningside Terrace, Edinburgh, EH10 5HF UK; 3 National Autistic Society, North East Resource Centre, Newcastle upon Tyne, NE5 2LW UK; 4 Population Health Sciences Institute, Newcastle University, Level 3, Sir James Spence Institute, Royal Victoria Infirmary, Queen Victoria Road, Newcastle upon Tyne, NE1 4LP UK; 5 Salvesen Mindroom Research Centre, Child Life and Health, Royal Hospital for Sick Children, Edinburgh, EH9 1LF UK; 6 Royal Edinburgh Hospital, Kennedy Tower, Morningside Terrace, Edinburgh, EH10 5HF UK; 7 Durham Research Methods Centre, Durham University, South Road, Durham, DH1 3LE UK; 8 Population Health Sciences Institute, Newcastle University, Baddiley-Clark Building, Richardson Road, Newcastle upon Tyne, NE2 4AA UK; 9 Department of Anthropology, Durham University, South Rd, Durham, DH1 3LE UK; 10 Newcastle Clinical Trials Unit, Newcastle University 1-4 Claremont Terrace, Newcastle upon Tyne, NE2 4AE UK; 11 Psychology Department, Durham University, Science Laboratories, South Road, Durham, DH1 3LE UK; 12 Derwentside CAMHS, 192 Medomsley Road, Consett, DH8 5HT UK

**Keywords:** Autism spectrum disorder, Randomised controlled trial, Restricted and repetitive behaviours, Parent-mediated intervention

## Abstract

**Background:**

Restricted and repetitive behaviours vary greatly across the autism spectrum, and although not all are problematic some can cause distress and interfere with learning and social opportunities. We have, alongside parents, developed a parent group based intervention for families of young children with autism, which aims to offer support to parents and carers; helping them to recognise, understand and learn how to respond to their child’s challenging restricted repetitive behaviours.

**Methods:**

The study is a clinical and cost-effectiveness, multi-site randomised controlled trial of the Managing Repetitive Behaviours (MRB) parent group intervention versus a psychoeducation parent group Learning About Autism (LAA) (*n* = 250; 125 intervention/125 psychoeducation; ~ 83/site) for parents of young children aged 3–9 years 11 months with a diagnosis of autism.

All analyses will be done under intention-to-treat principle. The primary outcome at 24 weeks will use generalised estimating equation (GEE) to compare proportion of children with improved RRB between the MRB group and the LAA group. The GEE model will account for the clustering of children by parent groups using exchangeable working correlation. All secondary outcomes will be analysed in a similar way using appropriate distribution and link function.

The economic evaluation will be conducted from the perspective of both NHS costs and family access to local community services. A ‘within trial’ cost-effectiveness analysis with results reported as the incremental cost per additional child achieving at least the target improvement in CGI-I scale at 24 weeks.

**Discussion:**

This is an efficacy trial to investigate the clinical and cost-effectiveness of a parent group based intervention designed to help parents understand and manage their child’s challenging RRB. If found to be effective, this intervention has the potential to improve the well-being of children and their families, reduce parental stress, greatly enhance community participation and potential for learning, and improve longer-term outcomes.

**Trial registration:**

Trial ID: ISRCTN15550611 Date registered: 07/08/2018. Sponsor and Monitor: Cumbria, Northumberland, Tyne and Wear NHS Foundation Trust R&D Manager Lyndsey Dixon, Address: St Nicholas Hospital, Jubliee Road, Gosforth, Newcastle upon Tyne NE3 3XT, lyndsey.dixon@cntw.nhs.uk, Tel: 0191 246 7222

**Supplementary Information:**

The online version contains supplementary material available at 10.1186/s13063-021-05175-y.

## Introduction

### Background

Autism spectrum disorder (hereafter, ‘autism’) is a lifelong neurodevelopmental condition affecting 1–2% of the population, with profound impact on diagnosed individuals, families, and society [1, 2]. Restricted and repetitive behaviours (RRB) are one of two key behavioural domains required for a diagnosis of autism [3]. They include repetitive motor mannerisms, rigid adherence to specific routines, highly circumscribed interests and extreme responses to everyday sensory experiences. Some RRBs are reported by autistic adults to be enjoyable, functional and helpful. They may provide a basis for friendship and can also build areas of strength, supporting skill development and yielding employment opportunities [4, 5]. However, RRB may also be outward signs of anxiety or distress, deserving of attention and care from others [6]. In some cases, RRB in children can have a distressing and negative impact for the child and their family members [7]. RRB can interfere with learning opportunities, community participation, aspects of health such as sleep and nutrition, and deplete child, parent, and sibling wellbeing [8]. Examples observed by practitioners include an elaborate and rigid bedtime routine lasting 3 h or more, a restricted diet of only 5 different foods and a repetitive pattern of pinching members of the public.

Here we use the term ‘challenging RRB’ to describe behaviours that fit the diagnostic description of a restricted and repetitive behaviour and that cause significant challenges for the child and in daily family life. A recent meta-analysis indicates that parents of autistic children experience higher levels of stress than parents of children with other disabilities and that stress is highly correlated with the presence of challenging RRB [9]. The impact is greater if parents/carers are not able to access appropriate advice and support [6]. Challenging RRB may also incur direct risk of injury for the autistic child [6]. They can take up large amounts of time, interfere with the child’s ability to engage in everyday living activities (e.g. personal care, mealtimes) and reduce access to learning [10–14]. Challenging RRBs are associated with increased rates of disruptive behaviours and aggression to others, which can further isolate the child and family and contribute to negative stigma [15, 16].

Parents report they do not receive specific professional advice on how to recognise or understand their child’s challenging RRB [17]. It is therefore critical that parents are supported to understand the different forms, functions and impact of RRB. They need to be able to distinguish possible underlying reasons for RRB and to identify those RRB that have the potential for deleterious impact on their child. They require evidence-based, effective and efficient strategies to specifically address challenging RRB. Such interventions, if found to be effective, have the potential to improve the well-being of autistic children and their families, reduce parental stress, greatly enhance community participation and potential for learning and improve longer-term outcomes.

In the UK, the National Service Framework for Disabled Children and Young People and those with Complex Health Needs highlights the potential impact of care for parents of children with autism, and the need for effective and efficient, evidence-based parent training interventions [18]. The evidence base for the effectiveness of parent-mediated interventions for young children with autism has been reported [19]. However, to date, most autism-specific early intervention programmes focus on social communication and rarely consider challenging repetitive behaviours despite this area being a priority for parents [20–23]. Furthermore, a systematic review on effectiveness of treatments for RRB in ASD has established that strategies that involve understanding the behaviour were promising but lacked a sufficient evidence, as the majority of studies used single case designs and focused solely on stereotypy [21]. Stereotypy is one specific type of RRB that is also less likely to be perceived as challenging. A review of trial databases (USNIH and UKCTG) indicates no current behavioural intervention trial registered for RRB (2020). There is one recently completed trial (completed Feb 2019) that aims to use mobile technology to reduce stereotypy (repetitive vocal and motor behaviours) in children with autism and is thus not relevant to the current trial [24].

The Managing Repetitive Behaviours (MRB) parent group intervention and study protocol were developed jointly in partnership with parents of young children with autism initially through two developmental parent groups. The intervention was designed to help parents recognise, understand and manage their child’s RRB [25]. The development work was followed by a pilot feasibility and acceptability randomised controlled trial (RCT). The results of the feasibility and acceptability pilot indicated that the MRB intervention was both acceptable to parents and feasible to deliver through routine clinical services. If effective, this intervention has the potential to extend the range of early interventions available to meet the needs of young children with autism and their families, ensuring best use of therapeutic resources and reducing the risk that challenging RRB persist with significant long-term consequences for the child and family. However, before recommending that this parent group intervention is included within local community early intervention services, a fully powered clinical- and cost-effectiveness RCT is required to rule out harmful effects of the intervention and quantify any benefit. This study is a phase 3 superiority trial, with a parallel group design in which each family is randomised 1:1 to one of the two parent group interventions.

## Methods and design

### Study design

This study is a UK three-site, two-group RCT. After consent families will be randomised at each site to receive either the 8-week MRB parent group intervention or the 8-week Learning About Autism (LAA) parent group equivalent to best current practice and will act as an attentional control for time and attention. Assessments are administered on entry (baseline) to the trial, at the end of the eight parent group sessions (10 weeks), at 24 weeks primary endpoint and at the 52 weeks follow-up.

### Study aims

The aim of this study is to evaluate the clinical and cost-effectiveness of the Managing Repetitive Behaviours (MRB) parent group intervention for young children with autism compared with a parent autism psychoeducation group, at 10, 24 and 52 weeks follow-up.

The clinical effectiveness of this intervention will be assessed by measuring whether children show an overall improvement in global functioning (primary outcome) at 24 weeks (primary endpoint) after the MRB intervention compared to the children whose parents had attended the LAA group. There are a number of secondary outcomes (measured at the end of the intervention and at 24-week follow-up), these include whether (i) parents increase their knowledge and skills in understanding and managing their child’s challenging RRB; (ii) children show a reduction in challenging RRB, and change in participation in everyday activities, parent stress and impact on family life; (iii) the intervention provides value for money for the NHS; and (iv) these improvements are maintained at 1 year from baseline.

We have carefully selected end of group and follow-up (24 and 52 weeks) outcome measures to capture both independent (blinded researcher), teacher and parent-reported changes. These include blinded ratings of improvement in child’s overall functioning (assessed using Clinical Global Impressions–Improvement (CGI-I)) and change in challenging RRB. Parent questionnaires will also provide information on child RRB, child participation and daily living skills, parent stress, self-efficacy and impact on family life. Parents will be aware of group status but teachers and the primary outcome assessor will be blind to group allocation.

We will evaluate maintenance of effect at the 52-week follow-up appointment which will also allow us to investigate any potential longer-term (downstream) impact on child and family functioning (e.g. social participation).

### Study setting

The MRB and the LAA parent groups will all take place in community settings in different geographical locations across the three UK sites: in Tyne and Wear, The Lothians and Teesside. This is in line with the UK National CAMHS Review (2008) [26], which indicated young people and families want accessible services in convenient places.

### Managing Repetitive Behaviour (MRB) intervention

MRB is a manualised intervention designed to help parents of young children with ASD to recognise, understand and learn how to manage their child’s challenging RRB. It is an 8-week manualised intervention designed to be delivered by community-based professionals with knowledge and experience of working with young children with ASD and their families who have been trained to deliver the MRB intervention. Each weekly session lasts for approximately 2 h (total duration of group intervention is ~ 16 h).

Each parent will be provided with a manual, related weekly materials and individualised support to identify strategies to address challenging RRBs. ‘At home’ activities will be set each week for parents and children to complete between sessions. The opportunity to identify and practice new ideas outside of the clinic setting is important for investigating how best to generalise the approach into different settings for each child and family.

Parents have the opportunities for individual support and group learning. In weeks 2 and 7, they meet the group leaders individually to select a target challenging RRB to focus on during the group and to practise the new skills they are learning, thus ensuring that strategies are individually tailored for each child and are developmentally appropriate.

### Learning About Autism (LAA) parent group

LAA is an 8-week manualised parent psychoeducation group that will act as an attentional control. LAA is designed for parents of young children with autism and focuses on understanding what autism is, and provides support for parents of young children with autism. It is designed to be run in the community with professionals who have experience of working with young children with autism and their families, and have been trained by a UK-based autism charity, the National Autistic Society, to deliver the intervention. Each weekly session lasts for approximately 2 h (total duration of group intervention is ~ 16 h).

The groups will offer parents and carers psychoeducation on understanding autism and what that means for their individual child such as helping them understand their child’s social communication difficulties and behaviour in different environments. It will also provide advice and guidance on strategies and approaches for dealing with behaviour problems in young children with ASD. There will be opportunities for mutual support and sharing of ideas with other parents. Thus increasing parents’ and carers’ understanding, confidence and responsiveness to their child’s patterns of communication and interaction. This is equivalent to current best practice. It will not include any specific information about the role and functions of RRB, functional analysis and tailored strategies to manage challenging RRB.

### Recruitment and eligibility criteria

Families will be identified via local community National Health Service (NHS) clinical services. Clinicians are asked to introduce the study to families fitting the inclusion criteria, discuss the study with them and if interested give them study packs. For some clinical services where children may not be seen as frequently, postal study packs will be sent to identified families. Interested parents are asked to complete and return to the research team an expression of interest form. The research team will then contact parents they are interested in hearing more about the study to arrange a face-to-face meeting to discuss the study, what participation involves, answer any questions they may have, and obtain written informed consent.

Parents are eligible for study entry if their child meets the following criteria: (1) aged 3–9 years 11 months at the time of consent with a clinical diagnosis of autism or autism spectrum disorder; (2) experience challenging RRB. Parents themselves will have (1) sufficient spoken and written English to provide written informed consent, complete assessments, and participate in the intervention; (2) are willing to be randomised and attend all group sessions for the allocated arm of the study; and (3) agree to maintain their child’s current medication regime until the primary 24-week end point (clinician changes to medication are permitted). Parents are not eligible for study entry if their child meets the following criteria: (1) no clinical diagnosis of autism or ASD; (2) no challenging RRB (3) is currently taking part in another parent group based intervention trial; or (4) has a sibling already taking part in this study. Furthermore, children of parents who have significant mental health difficulties will not be eligible to participate. There will be no special criteria for discontinuing or modifying allocated interventions.

### Training and fidelity

All parent group sessions in both arms of the trial will be recorded, both to facilitate opportunities for supervision of the group leaders and to provide access to a random sample of recorded sessions across all sites for the independent evaluation of (i) the fidelity to the delivery of the intervention and (ii) the therapeutic competence of the individual group leaders’ delivery of this manualised intervention. Fidelity will be formally assessed by independent raters watching 10% of randomly selected tapes and utilising the MRB Fidelity Rating Scale as developed during the pilot feasibility and acceptability study with adaptations for the content of the LAA parent groups.

At the start of the research, the group leaders and site clinical leads will attend a 1 day MRB training course learning how to use the training manual and the materials for each 2 h weekly session and planning for homework tasks. Each group leader will learn about group processes and gain skills to deliver the manualised intervention in a participatory style, in combination with strategies to personalise the programme for each parent. The senior trainer (LD) alongside the chief investigator (VG) will deliver the introductory training course. LD will also visit each site as each new group is started. Furthermore, to ensure the intervention is being delivered to the families as intended with high fidelity to the manual, the senior trainer will alongside the local site clinical lead, will undertake weekly supervision of group leaders at each site. Group Leaders delivering the LAA parent group will attend a 1-day training course specifically designed for this study to ensure they are trained in the skills needed to deliver the programme. This course has been designed by the National Autistic Society and will be delivered by DG.

Parent attendance at group sessions will be recorded and monitored. Parents who miss a parent group session will be contacted by a group leader to provide a brief catch up of session and handouts for that session will be posted out. Therapy compliance and receipt of other interventions outside of the protocol will be monitored.

### Avoidance of contamination

There will be separate clinical and research leads at each site and separate training and supervision structures. Researchers will be located separately from staff involved in delivery of the MRB and LAA intervention. Research interviews and assessments will be conducted mostly at home visits, with the Autism Diagnostic Observation Schedule-2 (ADOS-2) being undertaken at University sites. All parents will be reminded prior to every contact with research staff not to talk about group allocation. Furthermore, we will also consider issues around potential contamination of the active attentional control LAA group, by ensuring the LAA families are not on the case load of the MRB intervention group leaders. The clinical and research leads in the trial are experienced in studies of this type and therefore able to take steps to discern any potential risk of contamination bias and avoid it.

### Baseline characterisation and outcome measures

#### Baseline characterisation measures

The following baseline measures will be collected:

- Demographics: a bespoke demographics tool has been designed for the study.
- Autism Diagnostic Observation Schedule-2 (ADOS-2 [27];) is an observational assessment undertaken by a trained researcher. It is a semi-structured set of play and social communication activities. ADOS-2 has demonstrated good inter-rater reliability coefficients (Fleiss’ ĸ was .38 (range .19–.55) and Cohen’s ĸ was .69 (range .61–.76) for all modules) in the clinical setting [28].
- Social Responsiveness Scale – Second Edition (SRS-2 [29];): is a 65-item questionnaire measure of social and communication features The pre-school and school-age form has demonstrated adequate inter-rater reliability across parent and teacher ratings. Correlations were .77 and .61, respectively. There is strong consistency across items on the SRS-2 school age form which yielded a total reliability coefficient of 0.95 [30].

### Child outcome measures

#### Primary child outcome measure

- Clinical Global Impression-Improvement Scale (CGI-I) [31] provides a standardised framework to assess how much behaviour has improved or worsened relative to the child’s baseline state using a 7-point scale. Research demonstrates that the interclass correlation coefficient (ICC) between therapist and patients rating of CGI-I was 0.65 indicating a moderate to high agreement between the therapists CGI-I rating and patient’s perspective on their condition [32].

#### Secondary child outcome measures

- Target Behaviour Vignette [33]. As part of the baseline assessment, we require parents to undertake a detailed semi-structured interview during which they are required to identify two challenging RRB. The protocol was developed by the Research Units on Paediatric Psychopharmacology (RUPP) Autism Network [33] and has demonstrated high levels of agreement between expert raters. Arnold et al. [33] reported an Intraclass Correlation Coefficient (ICC) of 0.895 across a panel of five raters.
- Repetitive Behaviour Questionnaire - 2 (RBQ-2) [34] The RBQ-2 is a 20-item questionnaire that measures the frequency and intensity of RRB. RBQ-2 demonstrates good internal consistency for RRBs for children with autism from 2 to 17 years. The internal consistency was high for the Total RBQ-2 scale (Cronbach’s alpha = 86 for 20 items and .86 for 19 items) [35].
- Teacher Repetitive Behaviour Questionnaire 2 (Teacher RBQ-2) [36]. This is the corresponding version of the parent RBQ-2 for completion by teachers/teaching staff.
- Vineland Adaptive Behaviour Scales3 (VABS 3) [37]. The VABS 3 measures aspects of the child’s level of adaptive functioning. VABS 3 has demonstrated excellent internal consistency (Cronbach’s alpha 0.90 to 0.98) and content, construct, and concurrent validity [37]. It has been used in numerous autism studies.

#### Economic outcome measures

##### Primary economic outcome measure

- Incremental costs to achieve target difference in the CGI-I at 24 weeks: The improvement scores from the CGI-I will be taken from each randomised arm of the trial to inform the efficiency of the intervention. A cost per incremental improvement of CGI-I scale will be calculated in each pathway.

##### Secondary economic outcome measures

- Costs to the family: Cost to the family related to MRB will be estimated. Resources questionnaires and time and travel cost questionnaires are used to aid the estimation of these costs. Total cost to the family will be reported at 24 and 52 weeks.
- Incremental cost per QALY gained for the child: The CHU9D [38] will be measured in both arms of the trial to measure quality of life in relation to the child. This will be measured at baseline, 24 and 52 weeks. The CHU9D proxy version will be used [38]. The CHU9D is a paediatric generic preference-based measure of health-related quality of life that is suitable for use in this particular patient group. This measure has an acceptable level of internal consistency (Cronbach alpha 0.781) [39]. Following recommended practice parents/caregivers will be asked to complete the CHU9D with the child at the three time points. The responses from this instrument will be used to create utility values, which will be incorporated in QALY outcomes. This will be expressed an in an average incremental costs per QALY ratio for the children in each arm.
- QALYs for the caregiver: The EQ-5D-5L [40] will be completed at baseline, 24 and 52 weeks by the caregiver for the child. The EQ-5D-5L is a standardised instrument for use as a measure of health outcome. It is applicable to a wide range of health conditions and treatments. The EQ-5D-5L health questionnaire provides a simple descriptive profile and a single index value for health status. Initial psychometric properties of the 5-level EQ-5D show that results for test-retest reliability for average ICCs is 0.69 and VAS 0.51 indicating moderate reliability [41]. The responses from this instrument will be used to create utility values, which will be used to create QALYs for the caregivers. This outcome will be included as part of the cost consequence analysis.
- Cost-consequences: A number of primary and secondary clinical outcomes, quality of life effects for the child and quality of life effects for the caregivers will be used as outcomes for the cost-consequences analysis.
- Use of the health care resources. Resources which are used by the children will be measured using a bespoke questionnaire. Parents/caregivers will be asked to report the amount of times that their child with ASD accesses certain services (e.g. GP or outpatient appointments). This information will be used to calculate the average cost of services for each intervention.

### Family outcome measures

- The Autism Family Experience Questionnaire (AFEQ) [7]. This questionnaire measures broader impact of an intervention on the family. Parent (Cronbach’s alpha 0.85), Family (0.83), Child Development (0.81), Child symptoms (0.79), AFEQ total (0.92) [7].

### Secondary parent outcome measures

- Parent self-efficacy [42]: This 15-item self-report questionnaire that rates parental confidence in managing behaviours on a 6-point scale ranging from 0 (no confidence) to 5 (complete confidence).
- Autism Parenting Stress Index (APSI) [43]. This is a measure of parenting stress specific to core and co-occurring features of autism. The overall APSI scale score demonstrates acceptable internal consistency (Cronbach’s alpha 0.83) and test-retest stability (Cronbach’s alpha 0.88) for parents of children with autism [43]. Psychometric properties are good (e.g. Cronbach’s alpha 0.83).
- Warwick-Edinburgh Mental Wellbeing Scale (WEMWBS) [44] is a 14-item general population screen of wellbeing and is psychometrically robust with good internal consistency (Cronbach’s alpha 0.89) and test-retest reliability (ICC 0.83).

### Participant timeline

#### Procedures

##### Data collection

Baseline assessment and follow-up measures will be collected by RAs blinded to the outcome of randomisation. RAs are trained to high levels of reliability in all baseline characterisation and outcome measures (see Table 1). All families will be allocated a unique number that will be used to identify them on all paper assessment forms throughout the trial. All data collected on paper will be inputted into a data management system for statistical analysis and all identifying data will be stored securely separately. The Clinical Data Management System (Elsevier’s MACRO) used for this trial is fully compliant with all regulatory frameworks for research of this nature. Patients cannot be identified from eCRFs. The CI or delegated person will monitor completeness and quality of data recording in eCRFs and will correspond regularly with site PIs (or their delegated team member) with the aim of capturing any missing data where possible and ensuring continuous high quality of data. All study data will be treated in accordance with the latest Directive on Good Clinical Practice (2005/28/EC).

**Table 1:** Time points at which measures and data are collected

Procedure	Screening	Baseline	Treatment phase	Follow-up		
			Weeks 1–8	Week 10	Week 24	Week 52
Informed consent	X					
Child and parent demographics*	X				X	X
Eligibility	X					
ADOS-2		X				
SRS-2		X				
CGI-I					X	
RBQ-2		X		X	X	X
Teacher RBQ-2		X		X	X	X
Measurement of the target behaviour vignette		X		X	X	X
VABS 3		X			X	
Parent self-efficacy questionnaire		X		X	X	X
Autism Parenting Stress Index		X		X	X	X
WEMWBS		X			X	X
Autism Family Experience Questionnaire		X			X	X
Proxy completed CHU9D		X			X	X
EQ-5D-5L		X			X	X
Resource use questionnaire		X			X	X
Time and travel questionnaire		X				
Randomisation**		X				
Weekly intervention (MRB or Learning About Autism)			X			

*Child demographics to include child age, gender, type of nursery/school, diagnosis, current medications, additional diagnoses, ethnicity and previous intervention exposure

*Parent demographics to include level of education, employment status, family structure, attendance at previous courses or interventions relating to children with a diagnosis of ASD

**Randomisation to take place following completion of baseline assessment

Randomisation will be done through the Sealed Envelope (www.sealedenvelope.com/) web-based randomisation service. Allocation will be by a minimisation scheme instead of stratified randomisation to minimise sample fragmentation because of the number of strata and to avoid accidental imbalance between the MRB group and the LAA group. Child level randomisation was preferred controlling for child level characteristics that could affect the primary outcome; treatment centre, age strata (under 5th birthday, 5th birthday and older), ethnicity, and gender. Each case will be assigned a participant ID number and treatment allocation emailed separately to the clinical leads at each site. The clinical leads will inform the families of the outcome of randomisation and this will also be recorded in the participant’s NHS patient record.

We will collect information about severe adverse events; as well as recording severe adverse events in a standard format, we will include events of special interest particularly relevant to this trial, such as significant changes in family or school situation.

### Data management

A Data Monitoring Committee (DMC) will meet once a year to receive reports on recruitment and severe adverse events. The DMC will evaluate the findings of the internal pilot and submit report to NIHR, independently chaired and with an independent statistician. Severe adverse events, and actions taken, will be logged by the senior trial manager (CTU) and a report presented to DMC. The RA will discuss challenges with data collection with research leads at each site and the core research team. The RAs will be responsible for ongoing review of data completeness and any concerns will be discussed within the core research team, CTU and the Sponsor as appropriate.

Primary, secondary and relevant exploratory and sensitivity analyses of the data will take place by the trial statistician and health economist in collaboration with the Chief Investigator. The senior statistician will independently reproduce the primary analyses whilst still remaining blinded to the intervention groups. The senior statistician will have an overview of the entire analyses and will explicitly check the statistical codes. Other members of the team (e.g. the trial health economist) will also have access to data and will undertake analysis as appropriate and necessary. We have carefully considered the ethical implications in relation to this type of parent group intervention trial and there are no anticipated detrimental issues to participants. There are therefore no planned interim analyses. Any arrangements for other researchers in the general field to have access to the primary data will be negotiated separately and COREC informed.

### Statistical analysis

#### Sample size

We plan to approach approximately 325 families and expect to randomise 250 families (125 randomised to each arm). Assuming 5% type I error, 90% power, 10% intra-group correlation and equal allocation ratio, 224 families (an average of 8 families per parent group) are required to detect 20% improvement rate between the MRB intervention and Learning About Autism group at 24 weeks. Allowing for an attrition rate of 12%, 250 families will be randomised. The 10% intra-cluster correlation was based on review of group interventions in education trials [45]. Sample size was calculated in R using n4pros in CRTSize package [46].

### Analysis plan

A statistical analysis plan will be written and agreed by the Trial Steering Committee and Data Monitoring Committee before any analysis is undertaken. All statistical analyses will be carried out using the latest version of R software [46]. All analyses will be done in accordance with intention-to-treat principle where all children and parent outcomes are analysed as randomised. In accordance with CONSORT statement for non-pharmacological interventions, we will report all participant flow. Data will be summarised by trial arm. N, Mean ± SD, (or median ± IQR if data are skewed), Minimum and Maximum will summarise continuous variables, whereas number and percentages will be used to summarise categorical variables.

The analysis of the primary outcome at 24 weeks will use generalised estimating equations with binomial distribution and logit link. Exchangeable working correlation will be used to account for the clustering of children by parent groups. The continuous secondary outcomes will first be analysed at 24 weeks using a difference-in-difference model based on linear mixed effect model accounting for paired data (at baseline and at 24 weeks) per child and clustering of children by parent groups. The same model will be applied to the data at week 52, which will be analysed as longitudinal data incorporating data at baseline and 24 weeks. All binary or categorical secondary outcomes will be analysed using generalised estimating equations. We will also perform subgroup analysis and safety analysis, sensitivity analysis for missing data and assess the impact of the COVID-19 pandemic on primary and secondary outcomes.

### Health economics

#### Economic evaluation

The economic evaluation will be carried out from the perspective of the NHS and personal social services. A cost-effectiveness analysis within trial which will compare the costs to achieve the target mean difference in the CGI-I in both the MRB and Learning About Autism groups at 24 weeks. A cost-utility approach will also be undertaken using the data from the CHU9D questionnaire to synthesise QALYs for the children and compare the interventions using an incremental costs per QALY approach. To measure the benefits which would not be captured in the metric of a QALY. Finally, a cost-consequence will be used to compare costs and benefits from a wider perspective (for example the broader costs to families).

#### Measurement of effects

For the cost-effectiveness analysis, the effectiveness measure will be based on the results from the primary trial outcome; the target mean difference in the CGI-I.

The costs utility analysis will use the responses from the CHU9D based on the proxy responses from child’s caregiver. The CHU9D will be administered at baseline, 24 weeks and 52 weeks. This will measure the quality of life of the child which will be converted into QALYs for each child using the under the curve approach and an average incremental cost per QALY in each randomised arm will be calculated.

The caregiver will complete ED-5D-5L at baseline, 24 and 52 weeks. These responses will measure quality of life in relation to the caregiver and will be scored using the values sets for England. This data will also be converted into QALYs using the under the curve approach. The QALYs which are calculated for the carers will be included as part of the cost-consequence analysis. Further consequences will be examined as part of the cost consequences analysis including primary and secondary clinical outcomes, particularly the health-related quality of life of the child and their care-giver. These will include benefits which cannot be included with the scope of the QALY outcome.

#### Analysis

For the cost-effectiveness analysis, an incremental cost per unit change in the CGI-I scale will be calculated, with the aim of calculating the cost for achieving a minimally important difference in the CGI-I. Point estimates of costs and effects, cost effect plots and acceptability curves will be produced. Statistical imprecision and uncertainty will be examined using a stochastic sensitivity analysis. The cost-utility analysis will be analysed in a similar way, to the cost-effectiveness analysis. A formal decision analytic model is not currently planned but may be used if the cost of the intervention is not offset by a reduction in resource use or gain in QALYs for the child. If the results are conclusive (i.e. the intervention more effective and less costly or less effective and more costly) then a model will not be required.

The cost-consequence analysis will present the costs and consequences as a difference between randomised groups with appropriate measures of variance.

### Monitoring

#### Data monitoring and ethics committee

The project has a Trial Steering Committee (TSC) independently chaired by Professor Patricia Howlin (Emiritus Professor of Clinical Child Psychology) and is comprised of a panel of independent members including a Statistician, Health Economist and two Parent Representatives as well as Non-Independent members that form the Trial Management Group (TMG). The Data Monitoring Committee (DMC) is independently chaired by Professor John Jerrim (Professor of Education and Social Statistics) and comprised of a panel of Independent members including a Principal Clinical Psychologist and Post-Doctoral Research Associate as well as Non-Independent members that are part of the Trial Management Group. Serious adverse events, and actions taken, will be logged by the senior trial manager (CTU) or trial manager (CTU) and a report presented to DMC. The DMEC is independent of sponsor and funder and declares no competing interests. Further details of the DMEC charter are available from the trial manager.

##### Recording and reporting AEs and SAEs

For the purposes of this trial, only serious adverse events (adverse events which meet the criteria for seriousness) will be captured for the parent/carer and child participants. Serious adverse events will be captured from the start date of intervention until the follow-up assessment at week 24.

##### Events of special interest

As well as collecting and ensuring SAEs are reported, events of special interest will also be collected. An event of special interest is any event relating to child wellbeing and family/life difficulties which is not expected and not anticipated in ‘normal day-to-day life’, but is not a physical medical event. Events of special interest will be recorded for both the parent and child participants from the start date of the intervention until the follow-up assessment at week 24.

### Dissemination

The dissemination strategy for this research will include several complementary strands of activity. We will work closely with parents and the autism community to ensure that the results are interpreted and reported in a meaningful way: The results of the study will be shared as follows:

1. Local dissemination at each site including parent newsletters
2. Wider national and international dissemination including conferences and publications
3. NHS Clinicians and Commissioners events to discuss the implications of the research

## Discussion

The design will take a rigorous scientific approach by utilising a three-site, two-group randomised controlled design comparing two active parent group interventions. This study will evaluate the clinical and cost-effectiveness of the Managing Repetitive Behaviours (MRB) parent group intervention compared with the Learning About Autism psychoeducation parent group. The Learning About Autism (LAA) parent group, delivered by staff trained by The National Autistic Society, and equivalent to best current practice, allows us to control for the non-specific social group benefit of mutual sharing of experience and support between parents.

Parent group based interventions provide opportunities for mutual learning and sharing of ideas, allowing parents to discuss how best to support their child’s development. This fosters opportunities for parents to learn from and support each other, in turn building parents’ knowledge and confidence to support their child’s needs. However, in our MRB parent group intervention, we have included targeted support on challenging restricted and repetitive behaviours that are interfering in a deleterious way for the child. There is some information on restricted and repetitive behaviours in the LAA parent group but MRB focuses more on assisting parents to understand the role that RRB may have in their autistic child’s life. In this way, we anticipate that MRB will have a greater beneficial effect on reducing identified challenging RRB and improving child overall wellbeing.

Moreover, we will evaluate whether providing parents with the skills to effectively understand their child’s challenging RRB has a greater effect on parental wellbeing, sense of competence, reducing stress and improving family cohesion than a general autism psychoeducation parent group. In this way, the results from this RCT have the potential to help autism researchers explore the active ingredients of clinical trials.

We have carefully considered in collaboration with parents and professionals the utility of the outcome measures chosen to assess the effects of the intervention on challenging RRB, overall child functioning and parent and family wellbeing. We have taken a rigorous approach to outcome assessment with independent objective measures, where possible. We are aware of limitations in autism research from over reliance on unblinded parental report measures and have therefore included other outcome measures such as teacher-rated outcomes.

If found to be effective and efficient in the proposed evaluation study, this early parent group based intervention targeting challenging RRB has the potential to fill an identified but unmet need and thus improve the wellbeing of autistic children and their families, reduce parental stress, greatly enhance community participation, increase learning opportunities and improve longer-term outcomes.

### COVID-19 addendum

Following the announcement from the government on 23rd March 2020 in response to the COVID-19 pandemic, a strict UK lockdown was imposed, with implications for all clinical services and research trials across the UK. For our Managing Repetitive Behaviours (MRB) randomised controlled trial (RCT) this has meant a great deal of innovative creative thinking to design a revised research protocol for the conduct of all aspects of the research and the delivery of the two parent group based interventions. The aim was to ensure that recruited families and all the clinical and research staff were safe and that all our procedures were compliant with national government guidance documents and the required health and safety recommendations and procedures from the National Institute for Health Research (NIHR) and the Department of Health. With support from the research sponsor and funder an agreed plan to do assessments remotely, and run parent groups online was made.

A key priority for our clinical research team was to continue to keep the study open, to maintain as best as we could the integrity of the study and retain recruited families to the end of the trial despite the restrictions imposed by the COVID-19 pandemic. We are aware that families of autistic children are facing additional challenges in light of COVID-19 with reduced access to support and services. This was an added reason to remain in contact with families recruited to our study. We heard from parents that many children are finding changes to their usual routines very challenging and that there was a disproportionate impact on families caring for autistic children. This is important information that may impact on the conduct and results of this RCT. For all these reasons we were very keen to keep in touch with our recruited families for the duration of these challenging times. However, we also needed to take proactive precautions to protect the health of all the families in the trial and our staff in line with recommendations from the government, NHS and NIHR and comply with local site restrictions, which varied between sites. The trial team have worked with all sites, sponsor and funder to ensure the most appropriate actions have been taken.

The Trial Management Group with sponsor support on 26th March 2020, agreed all study activities would be performed remotely by sites wherever possible. Following this, we implemented non-substantial amendment 08 (NS08) on 3rd April 2020 as sponsor category C to reflect remote working changes with protocol version 06.

#### COVID-19 remote working measures

1. Study Research Associates (RA) are now working remotely at home in line with local policies following government advice and restrictions. Investigative site files have been re-located to clinical sites wherever possible; however, access to these and any paper source documents is now either limited or not possible at this time.
  - RAs working remotely at home must have access to secure laptops, which connect to the University/NHS server as appropriate. Phone calls to parents can be made at home but no patient identifiable data can be stored in homes.
  - All new paper source documents, including any RA notes, will either be recorded electronically for this period of time so that no patient details are stored outside of the local site server, or written on paper copies with no participant Identifiable data (PID) where applicable.
  - Remote supervision of RAs to be arranged at each site, with local principle investigator (PI) oversight, and documented.
2. Ongoing review of the situation and ability to remain open to any study activities
  - Sites to inform Chief Investigator (CI) and Newcastle Clinical Trial Unit (NCTU) of any changes in availability of staffing at each site
  - Study recruitment should be halted at individual site level if sites are no longer able to deliver the study. An amendment will only be submitted if the study is halted at sponsor level as discussed with the CI and NCTU.
  - To consider availability of the CI to provide central study advice.
  - The NCTU will inform sites of any overall study halt. It is then the responsibility of the local clinical team to risk assess participants that are already in the study.

A suite of documents including a contingency plan and working instruction have been created to aid all sites in the day to day workings of the study and remote working procedures. A sponsor specific contingency document has also been created to ensure sponsor is kept up to date with all the changes put in place. Furthermore, any future changes to the study relating to COVID-19 will be reviewed in line with the government guidance updates.

#### Intervention parent groups

We have explored digital options with our NHS clinical colleagues at each of the three sites to identify a secure and reliable platform that allows us to deliver our parent groups online. It was important the technology was easy to use for parents and group leaders, but still allowed group leaders to present information and participate in group chat with families.

For some families, attending online parent groups has been an easier option for them to consider as it has removed some of the barriers such as geographical, transport, work or other commitments that had previously prevented them from attending a parent group. We are mindful of issues of digital poverty but initial feedback from families has been positive, and most families have been able to join a parent group using a range of devices such as smart phones, tablets or computer.

### Trial status

Protocol Version 6.0; approval date: 02/04/2020.

The trial is recruiting. Recruitment began on the following dates for each site:

CNTW 02/10/2018; Lothian 20/11/2018; TEWV 22/11/2018.

Recruitment is due to complete on the 31/08/2020.

## Supplementary Information

**Additional file 1.** SPIRIT Checklist.

**Additional file 2.** Ethical approval documentation. HRA ethical approval.

**Additional file 3.** Copy of original funding documentation. NIHR funding documentation.

## Data Availability

All data in the trial will be anonymised. A central master file will be held by the trial manager at the Newcastle Clinical Trials Unit. This will contain the key linking anonymised trial name to personal details. This eCRF pack will be backed up securely within the web-based data entry service of Newcastle University CTU. All data will be entered into the Newcastle CTU web-based secure MACRO database which has a full audit trail and appropriate quality control will be carried out during the trial and before the database lock. Primary analysis of the data will take place in Durham University, by the trial statistician. The datasets generated and/or analysed during the current study are available from the corresponding author on reasonable request and COREC informed.

## Abbreviations

AE: Adverse event
AR: Adverse reaction
ASD: Autism spectrum disorder
CHU9D: Child Health Utility 9D
CI: Chief Investigator
eCRF: Electronic Case Report Form
EoI: Expression of interest
DMC: Data Monitoring Committee
GCP: Good Clinical Practice
GP: General practice
MRB: Managing Repetitive Behaviours
NCTU: Newcastle Clinical Trials Unit
NIHR-HTA: National Institute for Health Research – Health Technology Assessment
NHS: National Health Service
PI: Principal Investigator
R&D: Research & Development
RA: Research Associate
RCT: Randomised control trial
REC: Research Ethics Committee
RfPB: Research for Patient Benefit
RRB: Restrictive and repetitive behaviours
QALY: Quality adjusted life year
SAE: Serious adverse event
SAR: Serious adverse reaction
SOP: Standard operating procedure
USAR: Unexpected serious adverse reaction
TSC: Trial Steering Committee
