# Supplementary material for: A clinical and cost-effectiveness trial of a parent group intervention to manage challenging restricted and repetitive behaviours in young children with autism spectrum disorder: study protocol for a randomised controlled trial
Source: Trials. 2021 Apr 1;22:240. doi: 10.1186/s13063-021-05175-y (PMC8012746; doi:10.1186/s13063-021-05175-y)
Supplement: Supplementary file 2 — Additional file 2. Ethical approval documentation. HRA ethical approval. [file 13063_2021_5175_MOESM2_ESM.pdf]

NHS
Health Research Authority
South West - Cornwall & Plymouth Research Ethics Committee
Level 3
Block B
Whitefriars
Lewins Mead
Bristol
BS1 2NT

<u>Please note</u>: This is the favourable opinion of the REC only and does not allow you to start your study at NHS sites in England until you receive HRA Approval

20 August 2018

Dr Victoria Grahame
Consultant Clinical Psychologist, Hon Clinical Senior Lecturer and Clinical Lead (CNDS)
Northumberland Tyne and Wear NHS Foundation Trust
Complex Neurodevelopmental Disorder Service (CNDS)
Walkergate Park, Benfield Road
Newcastle Upon Tyne
NE6 4AE

Dear Dr Grahame

**Study title:**	**Managing Repetitive Behaviours: A clinical and cost effectiveness trial of a parent group intervention to manage restricted and repetitive behaviours in young children with Autism Spectrum Disorder.**
**REC reference:**	**18/SW/0173**
**Protocol number:**	**RES-17-032**
**IRAS project ID:**	**246595**

Thank you for your letter of 6th August, responding to the Committee's request for further information on the above research and submitting revised documentation.

The further information has been considered on behalf of the Committee by the Chair.

We plan to publish your research summary wording for the above study on the HRA website, together with your contact details. Publication will be no earlier than three months from the date of this opinion letter. Should you wish to provide a substitute contact point, require further information, or wish to make a request to postpone publication, please contact hra.studyregistration@nhs.net outlining the reasons for your request.

Confirmation of ethical opinion

On behalf of the Committee, I am pleased to confirm a favourable ethical opinion for the above research on the basis described in the application form, protocol and supporting documentation as revised, subject to the conditions specified below.

Conditions of the favourable opinion

The REC favourable opinion is subject to the following conditions being met prior to the start of the study.

You should notify the REC once all conditions have been met (except for site approvals from host organisations) and provide copies of any revised documentation with updated version numbers. Revised documents should be submitted to the REC electronically from IRAS. The REC will acknowledge receipt and provide a final list of the approved documentation for the study, which you can make available to host organisations to facilitate their permission for the study. Failure to provide the final versions to the REC may cause delay in obtaining permissions.

Management permission must be obtained from each host organisation prior to the start of the study at the site concerned.

Management permission should be sought from all NHS organisations involved in the study in accordance with NHS research governance arrangements. Each NHS organisation must confirm through the signing of agreements and/or other documents that it has given permission for the research to proceed (except where explicitly specified otherwise).

Guidance on applying for HRA and HCRW Approval (England and Wales)/ NHS permission for research is available in the Integrated Research Application System, at www.hra.nhs.uk or at http://www.rdforum.nhs.uk.

Where a NHS organisation's role in the study is limited to identifying and referring potential participants to research sites ("participant identification centre"), guidance should be sought from the R&D office on the information it requires to give permission for this activity.

For non-NHS sites, site management permission should be obtained in accordance with the procedures of the relevant host organisation.

Sponsors are not required to notify the Committee of management permissions from host organisations

Registration of Clinical Trials

All clinical trials (defined as the first four categories on the IRAS filter page) must be registered on a publically accessible database within 6 weeks of recruitment of the first participant (for medical device studies, within the timeline determined by the current registration and publication trees).

There is no requirement to separately notify the REC but you should do so at the earliest opportunity e.g. when submitting an amendment. We will audit the registration details as part of the annual progress reporting process.

To ensure transparency in research, we strongly recommend that all research is registered but for non-clinical trials this is not currently mandatory.

If a sponsor wishes to request a deferral for study registration within the required timeframe, they should contact hra.studyregistration@nhs.net. The expectation is that all clinical trials will be registered, however, in exceptional circumstances non registration may be permissible with prior agreement from the HRA. Guidance on where to register is provided on the HRA website.

It is the responsibility of the sponsor to ensure that all the conditions are complied with before the start of the study or its initiation at a particular site (as applicable).

Ethical review of research sites

NHS sites

The favourable opinion applies to all NHS sites taking part in the study, subject to management permission being obtained from the NHS/HSC R&D office prior to the start of the study (see "Conditions of the favourable opinion" below).

Non-NHS sites

The Committee has not yet completed any site-specific assessment (SSA) for non-NHS research site(s) taking part in this study. The favourable opinion does not therefore apply to any non-NHS site at present. We will write to you again as soon as an SSA application(s) has been reviewed. In the meantime no study procedures should be initiated at non-NHS sites.

Approved documents

The final list of documents reviewed and approved by the Committee is as follows:

Document	*Version*	*Date*
Covering letter on headed paper [Cover Letter]		03 July 2018
Evidence of Sponsor insurance or indemnity (non NHS Sponsors only) [Insurance]		02 August 2017
IRAS Application Form [IRAS_Form_03072018]		03 July 2018
Letter from sponsor [Sponsorship Letter]		11 June 2018
Letter from statistician [Letter from statistician]		21 June 2018
Letters of invitation to participant [Parent expression of interest form]	01	22 June 2018
Letters of invitation to participant [Education professional letter]	02	31 July 2018
Non-validated questionnaire [Baseline Demographics form]	01	22 June 2018
Non-validated questionnaire [Time and Travel Questionnaire]	01	22 June 2018
Non-validated questionnaire [Repetitive Behaviour Questionnaire (RBQ-2)]	01	22 June 2018
Non-validated questionnaire [Repetitive Behaviour Questionnaire (RBQ-2)-Teacher version]	01	22 June 2018
Non-validated questionnaire [Parent self-efficacy Questionnaire]	01	22 June 2018
Non-validated questionnaire [Service Use Questionnaire]	01	22 June 2018
Non-validated questionnaire [Target Repetitive Behaviours]	01	22 June 2018
Non-validated questionnaire [Autism Family Experience Questionnaire (AFEQ)]	01	22 June 2018
Other [Pilot qualitative feedback]		23 October 2016

Participant consent form [ICF]	02	31 July 2018
Participant information sheet (PIS) [PIS]	02	31 July 2018
Referee's report or other scientific critique report [Reviewers' questions]		
Research protocol or project proposal [Protocol]	02	25 July 2018
Summary CV for Chief Investigator (CI) [CV]		01 March 2018
Validated questionnaire [ADOS-2 Phrase speech]		
Validated questionnaire [ADOS-2 Fluent speech]		
Validated questionnaire [ADOS-2 Pre-verbal single words]		
Validated questionnaire [CHU9D (proxy version with guidance notes for under 5 years)]		
Validated questionnaire [CHU9D (proxy version)]		
Validated questionnaire [EQ-5D-5L Self-complete]		
Validated questionnaire [SRS-2]		
Validated questionnaire [VABS-II]		
Validated questionnaire [WEMWBS]		
Validated questionnaire [Autism Parenting Stress Index (APSI)]		
Validated questionnaire [EQ-5D-5L User Guide]		
Validated questionnaire [CGI]		

Statement of compliance

The Committee is constituted in accordance with the Governance Arrangements for Research Ethics Committees and complies fully with the Standard Operating Procedures for Research Ethics Committees in the UK.

After ethical review

Reporting requirements

The attached document *“After ethical review – guidance for researchers”* gives detailed guidance on reporting requirements for studies with a favourable opinion, including:

- Notifying substantial amendments
- Adding new sites and investigators
- Notification of serious breaches of the protocol
- Progress and safety reports
- Notifying the end of the study

The HRA website also provides guidance on these topics, which is updated in the light of changes in reporting requirements or procedures.

User Feedback

The Health Research Authority is continually striving to provide a high quality service to all applicants and sponsors. You are invited to give your view of the service you have received and the application procedure. If you wish to make your views known please use the feedback form available on the HRA website:
http://www.hra.nhs.uk/about-the-hra/governance/quality-assurance/

HRA Training

We are pleased to welcome researchers and R&D staff at our training days – see details at http://www.hra.nhs.uk/hra-training/

**18/SW/0173**	**Please quote this number on all correspondence**

With the Committee's best wishes for the success of this project.

Yours sincerely

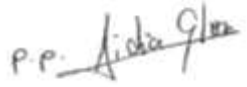

Canon Ian Ainsworth-Smith
Chair

Email:nrescommittee.southwest-cornwall-plymouth@nhs.net

Enclosures: "After ethical review – guidance for researchers"

Copy to: *Lyndsey Dixon, Northumberland, Tyne and Wear NHS Foundation Trust*
