# Supplementary material for: A clinical and cost-effectiveness trial of a parent group intervention to manage challenging restricted and repetitive behaviours in young children with autism spectrum disorder: study protocol for a randomised controlled trial
Source: Trials. 2021 Apr 1;22:240. doi: 10.1186/s13063-021-05175-y (PMC8012746; doi:10.1186/s13063-021-05175-y)
Supplement: Supplementary file 3 — Additional file 3. Copy of original funding documentation. NIHR funding documentation. [file 13063_2021_5175_MOESM3_ESM.pdf]

National Institute for Health Research

Evaluation, Trials and Studies Coordinating Centre
University of Southampton
Alpha House
Enterprise Road
Southampton
SO16 7NS

Tel: 023 8059 5586
Fax: 023 8059 5639

Email: hta.funding@nihr.ac.uk

7 September 2017

Dr Victoria Grahame
Consultant Clinical Psychologist/
Hon Clinical Senior Lecturer
Northumberland, Tyne and Wear
NHS Foundation Trust
Complex Neurodevelopmental Disorders Service
Walkergate Park Centre
Benfield Road
Newcastle upon Tyne
NE6 4QD

Dear Dr Grahame

16/111/95 - Managing Repetitive Behaviours: Clinical and cost effectiveness trial of a parent group intervention to manage restricted and repetitive behaviours in young children with ASD.

Following the HTA Funding Board in July 2017, and the budget review by the HTA Prioritisation Group, I am pleased to inform you that the HTA Programme has recommended funding for your above-titled project, subject to a satisfactory response to the Board's concerns, finance and IP questions detailed below. Please would you inform your co-applicants of this decision.

Please note that this award is not secure until the work has been contracted. Therefore, you must not publicise your achievement until you have received and signed a completed contract from us.

Please address the points raised below in a covering letter alongside an updated version of your full proposal and detailed project description.

Board Feedback

- The applicants should include clear and robust progression criteria for the internal pilot based on recruitment numbers and checking that key parameters are consistent with pre-trial assumptions.
- The applicants should consider the possibility of collecting data on patient's exclusion to identify the impact of the language barrier among potential participants.
- The Board felt that best practice should be included in the intervention arm as well as the comparator arm.
- The applicants will need to confirm that the research costs requested cover the study period.

Intellectual Property (IP) Feedback

- We would like to draw your attention to the draft contract, http://www.nihr.ac.uk/funding-and-support/funding-for-research-studies/manage-my-study/contracts-and-intellectual-property.htm and specifically to the background IP provisions. Please would you check what third party rights exist in terms of background IP. If there are none, please inform us,

as this means there will be no requirement for schedule C in the contract. If third party rights do exist in relation to background IP, please provide details so that they may be appended to the contract in Schedule C.

- It is NIHR's starting position that all arising foreground IP shall be held by the contractor. If you wish NIHR to consider alternative ownership arrangements, please provide details for consideration. The information you provide will feed into Schedule D of the contract.
- Consider what IP will be created by dissemination plans/materials. This study will produce IP, including data, which may inform guidelines. Although this may have no commercial value, there is potential patient benefit.
- Provide details of any third party organisations who will be providing the comparator/current best practice, along with further clarity on the arrangements reached.
- We note the intention to use the ADOS kit in the study. Please provide more information on:
  - Why the ADOS kit has been chosen and detail any other devices that were considered.
  - The process for choosing the ADOS kit.
- Confirm that licences are either not required, or have been sought for the use of the questionnaires.

Finance comments on Research and NHS Costs

Our Finance Department has raised the following queries relating to figures in your application form that you need to respond to:

- None of the co-applicant and staff costs are for the full 40-months of the project duration. Please confirm this is correct.
- The costs for the TBA 3 Durham appear high when salary and fte are compared. Please review this cost.
- Please provide a more detailed breakdown showing how the parent travel, RA travel for assessments, Durham University and Deborah Garland travel costs were calculated.
- Please confirm how many individuals will be attending international conferences.
- Are any of the ADOS costs associated with the intervention? If so, they should be included as NHS Treatment Costs.
- Please provide more data on the Clinical Data Management System.
- To ensure that the NHS costs are calculated correctly, please include in the NHS Treatment Costs section of form the costs for 125 parents receiving the parent psychoeducation group. In the NHS Usual Treatment Costs section, please include the costs for 250 parents receiving care/support as they would in current standard care.

If you require any additional advice about the attribution of costs, please contact the Support Costs team, email: nets-finance@nihr.ac.uk. Clarification of these costs is essential and they will need to conform to the DH guidance on costs, known as AcoRD, see: https://www.gov.uk/government/publications/guidance-on-attributing-the-costs-of-health-and-social-care-research .

We do not wish to slow down your project start-up but all these points will need to be addressed to the satisfaction of all concerned. The result of any discussions should be outlined in the response letter you send.

The open comments supplied by the peer reviewers concerning your proposal were sent to you to allow you to respond to their comments in advance of the board. These comments are available under 'view all my tasks' in the MIS and they may provide useful expert feedback as you prepare to begin your project.

Research Governance

HRA Approval is the process that covers research in the NHS in England. It replaces the need to make separate REC and R&D applications in England. HRA Approval comprises a review by a Research Ethics Committee and an assessment of regulatory compliance and related matters undertaken by dedicated HRA staff. It is being introduced during 2015. Information for applicants is provided on the HRA website at http://www.hra.nhs.uk/resources/hra-approval-applicant-guidance/. Details on the assessment that studies will undergo and the standards that they will be required to meet to receive HRA Approval are available here: http://www.hra.nhs.uk/resources/hra-approval-applicant-guidance/hra-assessment-criteria-and-standards.

Sponsors and applicants are strongly encouraged to familiarise themselves with the latest version of these standards. Information on the responsibilities of sponsors is provided at http://www.hra.nhs.uk/resources/before-you-apply/roles-and-responsibilties/sponsor.

Project Start Date

The National Institute for Health Research (NIHR) is committed to timeliness of research and rapid initiation of studies following funding board assessment. As part of work to help reduce delays in project set-up, the NIHR, HRA and DH agreed a change to the standard contract permitting release of payments in advance of receipt of ethical approval (see website for details).

We are keen to see this project get started and completed for the benefit of patients as soon as possible and you should start to prepare your protocol and be working towards gaining the necessary governance approvals required for you to start the project within a maximum of 8 months of receipt of this letter.

As a programme, we wish to act in a fair and reasonable way and understand that there are often multiple reasons for delays and some factors that are outside of your control. However, we anticipate that the study will have started within 8 months from the date of the letter, and as Chief Investigator for the study, you are responsible for ensuring the project starts in a timely fashion. We reserve the right to reconsider our offer of funding should you not start by this date.

Integrated Research Application System (IRAS) identification number

If your project will require ethical approval and/or any other governance approvals, please provide us with the IRAS identification number for this project in your letter of response to the Board, or justification of why it is not required for your project. Please note that you do not need to go through the full process of submitting an ethics request to obtain this; recent changes on the IRAS website http://www.myresearchproject.org.uk/ mean that you are able to obtain an IRAS identification number simply by registering your study. Collecting the identification number at this stage is part of a pilot to try to smooth the permissions and approvals pathway for researchers as part of the Health Research Authority's work.

Next Steps

Please log into your user account for the NETSCC Management Information System (NETSCC MIS) at https://netscc-mis.nihr.ac.uk to respond to the comments and requirements for changes. You will have access to the relevant parts of your application form to revise and resubmit with the required changes.

Additionally, as part of the submission, you should include a letter that responds to each comment point in turn and clearly explains your proposed action(s). You should also provide a revised copy of your detailed project description which shows all the amendments as 'tracked changes'.

Both these documents should be included as **PDF** uploads when submitting your revised application (please note that you should delete any old versions currently showing on the MIS before uploading the revised PDF versions).

Please ensure that we receive your response to the above feedback by **1pm on 5 October 2017**.

The post-award set-up process will be managed by **Rod Delanougerede** in the Monitoring team. Please direct all responses and future communications to him at netspostawardsetup@nihr.ac.uk or on 023 8059 5713**.**

Once the contract process is complete, the HTA Programme encourages successful applicants to consider submitting their protocols for review to journals such as the Lancet.

Yours sincerely

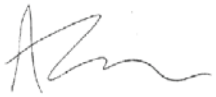

Alan Newman
Assistant Research Manager
E-mail: htacet@nihr.ac.uk
Direct Tel: 02380 597459

NIHR HTA Programme responsibilities on behalf of the Department of Health

1. To assess the scientific quality of the research as proposed.

2. To establish the value for money of the research proposed.

3. To consider the suitability of the research environment, particularly the experience and expertise of the chief investigator and other key researchers.

4. To ensure the sponsor takes on all appropriate responsibilities before the research begins.

5. To comply and co-operate with any necessary enquiry, audit or investigation.

6. To ensure that the study has ethical and any other necessary approvals before funding is released.

7. To ensure the appropriate indemnities are in place in line with contractual arrangements.

8. To appoint trial management committee chairs and members and their terms of reference.

9. To authorise and approve payments in line with contractual arrangements.

10. To work with the chief investigator to publish information concerning the trial and its findings in the HTA monograph, in line with contractual arrangements.

11. To assess progress, in line with contractual arrangements.

12. To authorise and record changes to protocol, in line with contractual arrangements.

13. To hold site visits and project initiation meetings when appropriate, in line with contractual arrangements.
